# The Effect of *Lacticaseibacillus paracasei* LPC100 and *Lactiplantibacillus plantarum* LP140 on Bone Mineral Density in Postmenopausal Women: A Multicenter, Randomized, Placebo-Controlled Study

**DOI:** 10.3390/jcm13195977

**Published:** 2024-10-08

**Authors:** Joanna Głogowska-Szeląg, Ilona Palka-Kisielowska, Wiesława Porawska, Joanna B. Bierła, Agnieszka K. Szczepankowska, Tamara Aleksandrzak-Piekarczyk, Bożena Cukrowska

**Affiliations:** 1 Department of Pathophysiology and Endocrinology, Silesian Medical University, Jordana 19, 41-808 Zabrze, Poland; szelag@poczta.onet.pl; 2 Specialized Medical Practice, Urbana 14, 40-750 Katowice, Poland; ilona-palkis@o2.pl; 3 Clinical Research Center, Śniadeckich 7/2, 60-773 Poznań, Poland; wporawska@reumedika.pl; 4 Department of Clinical Biochemistry, The Children’s Memorial Health Institute, Aleja Dzieci Polskich 20, 04-730 Warsaw, Poland; j.bierla@ipczd.pl; 5 Institute of Biochemistry and Biophysics, Polish Academy of Sciences, Pawińskiego 5a, 02-106 Warsaw, Poland; agaszczep@ibb.waw.pl (A.K.S.); tamara@ibb.waw.pl (T.A.-P.); 6 Department of Pathomorphology, The Children’s Memorial Health Institute, Aleja Dzieci Polskich 20, 04-730 Warsaw, Poland

**Keywords:** osteoporosis, postmenopausal women, probiotics, prevention, T-score, bone mineral density, vitamin D

## Abstract

**Objectives:** modulation of gut microbiota by probiotics has been proposed as a target for intervention to reduce bone mineral density (BMD) loss in the postmenopausal period. This study aims to evaluate the effect of *Lacticaseibacillus* (L.) *paracasei* LPC100 and *Lactiplantibacillus* (L.) *plantarum* LP140 on BMD in postmenopausal women in a multicenter, randomized, double-blind, placebo-controlled trial. **Methods:** the primary outcome was the change in T-score of the lumbar spine measured by dual-energy X-ray absorptiometry assessed after twelve-month probiotic supplementation. Secondary outcomes included changes in serological markers of inflammation and calcium–phosphate metabolism, body mass index, gastrointestinal symptoms, and satisfaction with the intervention. **Results:** a decrease in T-score indicating the progressive bone demineralization reached a statistically significant difference in the placebo group (from mean values of 0.06 ± 1.04 to −0.28 ± 1.12, *p* = 0.041) but not in the probiotic group (0.19 ± 0.99 and −0.08 ± 1.05, respectively, *p* = 0.125) with a *p*-value = 0.089 between the groups. No significant differences were found in secondary outcomes with the exception of vitamin D concentration and a significant reduction in some gastrointestinal symptoms in the probiotic group. A significant decrease in vitamin D level was observed only in the placebo group (*p* = 0.014). Probiotics were safe and well tolerated. **Conclusions:** this study suggests that the oral administration of *L. paracasei* LPC100 and *L. plantarum* LP140 may be a viable strategy to prevent BMD loss and vitamin D deficiency in postmenopausal women, but further research is necessary to confirm clinical benefits and to know the mechanism of action [ClinicalTrial.gov NCT06375668].

## 1. Introduction

Postmenopausal bone health is a significant concern for women due to hormonal changes, primarily the decline in estrogen levels during and after menopause. Estrogen drop leads to increased bone resorption resulting in a gradual loss of bone mass and a higher risk of osteopenia and osteoporosis—conditions characterized by low bone mineral density (BMD) and an increased susceptibility to fractures [1]. The World Health Organization (WHO) recognizes dual-energy X-ray absorptiometry (DXA) scans of the lumbar spine and/or hip as the gold standard for assessing BMD, which is reported as T-scores [2]. The T-score, measured in units of standard deviations (SDs), reflects the difference between the patient’s BMD and the mean BMD of healthy, young adult controls. According to the WHO, a normal BMD is within one SD of the control mean; T-scores between −1 and −2.5 indicate osteopenia, and scores below −2.5 indicate osteoporosis [3].

To maintain bone health in postmenopausal women, several strategies are recommended, including adequate calcium and vitamin D intake, weight-bearing and resistance exercises, and a healthy lifestyle without smoking and excessive alcohol consumption. A balanced diet rich in fruits and vegetables can also help preserve bone density during the postmenopausal period [3,4]. Recently, attention has turned to the gut–bone axis—the interaction between intestinal microbiota and the skeletal system—and its role in bone mineralization [5,6].

The gut microbiota, which are microorganisms colonizing the gastrointestinal tract, play a crucial role in human health. The number of gut-colonizing bacteria (10^14^ cells) is comparable to that of all cells in the human body and is ten-fold higher than the number of nucleated human cells [7]. The gut microbiota are considered as a “super organ” due to their roles in immune system development, intestinal barrier function, anti-inflammatory cytokine induction, vitamin production (mainly B vitamins and vitamin K), and digestion and absorption of nutrients, including minerals necessary for bone metabolism [8]. Experimental studies in germ-free (GF) mice have shown that the gut microbiota affect osteoclast activity leading to higher bone mass primarily due to osteoclastogenesis inhibition [9,10]. The intestinal microbiota also affect calcium absorption, facilitated by short-chain fatty acids (SCFAs) produced through bacterial fermentation [11,12]. Intestinal dysbiosis understood as disturbances in composition and functional activity of the gut microbiota, may lead to dysregulation of bone turnover [13,14].

Given the role of the gut microbiota in bone metabolism, modification of their com-position and activity has been considered as a preventive measure against osteopenia and osteoporosis [15]. Probiotics are live microorganisms that, when administered at adequate doses, confer health benefits to the host [16]. Probiotics can modify the gut microbiota composition and directly impact human health. A recent preclinical meta-analysis demonstrated that probiotics, especially species from the lactobacilli group (former *Lactobacillus* genus), have the potential to prevent postmenopausal osteoporosis [17]. However, clinical trials in postmenopausal women have not conclusively shown improvements in bone mineralization, highlighting the need for specific probiotic strains and further randomized, placebo-controlled trials [18].

This randomized, double-blind, multicenter, placebo-controlled study investigates the effect of a 12-month oral supplementation with a probiotic preparation containing *Lacticaseibacillus* (L.) *paracasei* LPC100 and *Lactiplantibacillus* (L.) *plantarum* LP140 from the NORDBIOTIC^TM^ collection on BMD, assessed via T-scores, in postmenopausal women. This study aims to demonstrate the protective effects of long-term administration of these specific probiotic strains in women with T-scores equal to or above −1.5 SD.

## 2. Materials and Methods

### 2.1. Study Design

This study was a randomized, double-blind, placebo-controlled, equal location ratio, parallel-group, multicenter clinical trial conducted from March 2022 to January 2024. This study was approved by the Bioethical Committee at the Silesian Medical Chamber (decision number 51/2021). This study was registered at ClinicalTrial.gov under the number NCT06375668.

**Figure 1:**
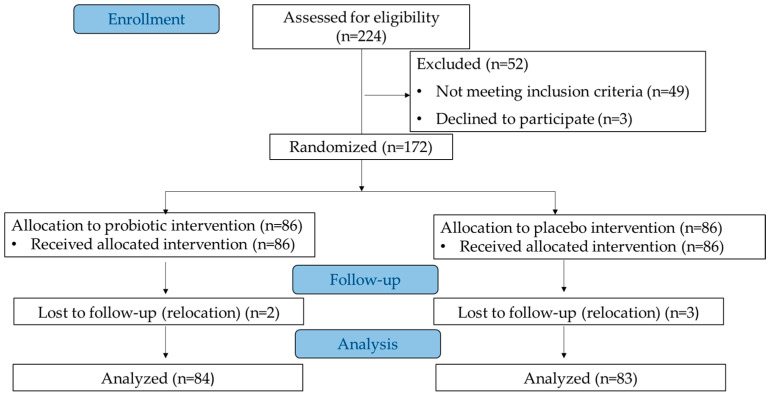
The study flowchart.

Out of 224 women screened, a total of 172 participants who met all the inclusion criteria and none of the exclusion criteria were randomized into two groups (probiotic or placebo) at a 1:1 ratio using a computer-generated randomization list from https://www.random.org/lists (accessed on 20 March 2022). The randomization was blinded to both patients and researchers. The intervention lasted 12 months, with the study schedule including the following visits: (i) a screening visit during which study participants were assessed by inclusion and exclusion criteria, (ii) randomization visit during which participants were randomly allocated to study groups and received a supply of the probiotic preparation or placebo; (iii) six clinic visits (after 2, 4, 6, 8, 10, and 12 months), (iv) five tele-consultations (after 1, 3, 7, 9, and 11 months). The study flowchart is presented in Figure 1.

Participants underwent (i) history-taking and physical examination at each clinic visit, (ii) DXA scans of the lumbar spine (L1–L4) at the screening visit and after 12 months, (iii) assessment of gastrointestinal symptoms (number of bowel movements per day, stool consistency, abdominal pain, and flatulence) at each visit and during tele-consultations, (iv) assessment of adverse events at each clinic visit and during tele-consultations, (v) body weight and height measurements, body mass index (BMI) calculation at the screening visit and after 6 and 12 months, (vi) peripheral blood collection during the screening visit and after 6 and 12 months, (vii) treatment satisfaction assessment using the Treatment Satisfaction Questionnaire (TSQ) after 2, 6, 10, and 12 months.

Stool consistency was assessed with the Bristol Stool Form Scale [19], and abdominal pain, nausea, and flatulence were assessed with a 5-point Likert scale [20]. Briefly, a score of 0 indicated no symptoms, and scores of 1–4 were based on the severity of symptoms; the higher the score, the more severe the symptoms. The TSQ consisted of 7 questions where 6 of them were rated on a 5-point scale, where 1 meant very negative and 5 meant very good, and the 7th question concerned the occurrence of side effects recorded as YES or NO. Blood tests included (i) complete blood count; erythrocyte sedimentation rate (ESR); serum high-sensitive C-reactive protein (CRP), calcium, phosphorus, and alkaline phosphatase levels (at the screening visit and after 6 and 12 months); (ii) serum vitamin 25 (OH) D levels (at the screening visit and after 12 months).

The safety of the probiotic product was monitored throughout the 12-month intervention. During the visits or tele-consultations, each patient was asked about adverse events, which were also assessed by the 7th question (Q7) to the TSQ.

### 2.2. Inclusion and Exclusion Criteria

Inclusion criteria were (i) postmenopausal women between 2 and 5 years after their last menstrual period with lumbar spine (L1–L4) T-scores measured by DXA equal to or above −1.5 units (SD), (ii) vitamin 25 (OH) D levels in peripheral blood serum equal to or above 31 ng/mL, and (iii) BMI between 18 and 30.

Exclusion criteria included estrogen and/or progestogen hormone therapy within the last 6 months; glucocorticosteroid or thyroid hormone therapy within the last 6 months; neoplastic disease; autoimmune disorders; malabsorption syndromes (including celiac disease); chronic kidney disease or kidney failure; endocrine disorders; diabetes mellitus and other chronic conditions affecting bone metabolism; antibiotic therapy; probiotic use and calcium or vitamin D supplementation within the last 2 months prior to study recruitment; treatment with antidepressant or antipsychotic agents within the last 3 months; substance use disorder (alcohol, drugs, and nicotine); history of organ transplantation; history of fractures; exposure to the SARS-CoV-2 virus within 14 days prior to study recruitment; an acute COVID-19 infection within 14 days prior to study recruitment; a surgical procedure scheduled to take place during the time frame of this study; participation in another study within the last 6 months; and inability to provide informed consent.

### 2.3. Intervention

Participants received capsules with a probiotic formulation consisting of *L. paracasei* LPC100 (DSM 33793) and *L. plantarum* LP140 (DSM 33804) at 5 × 10^9^ colony-forming units (CFUs) of probiotic bacteria in each capsule (Table 1). The placebo contained maltodex-trin. All capsules were identical in appearance and taste and marked as product A or B, provided in identical packaging with inscriptions containing the title of the study, the approval number of the Bioethical Committee, and the expiry date. Probiotics or the placebo were prepared, blinded, and delivered by Nordic Biotic Ltd. (Warsaw, Poland) and orally administered during the study at a dose of one capsule daily in the morning for 12 months.

### 2.4. Compliance

Participants received probiotics/placebo at each clinic visit and were asked to return the packaging and unused capsules at their next visit. Compliance was calculated based on returned capsules, with >90% compliance required for continuation in the study.

### 2.5. Outcomes

The main efficacy endpoint of the intervention was the change in T-score values from lumbar spine DXA scans assessed at the screening visit (baseline) and after 12 months.

Secondary efficacy endpoints included the changes in (i) biochemical parameter levels in the serum (calcium, phosphorus, vitamin D, alkaline phosphatase, CRP, and ESR), (ii) BMI values, (iii) severity of gastrointestinal symptoms, and (iv) treatment satisfaction assessment (TSQ).

### 2.6. Safety of Intervention

Adverse effects were monitored throughout the study during clinic visits and tele-consultations. Participants had continuous access to researchers via telephone.

### 2.7. Statistical Analyses

The sample size was calculated based on the previous study suggesting the difference in lumbar spine BMD T-score of 0.71 units between the placebo and probiotic groups with 95% of confidence interval in the probiotic group of –0.50 to 0.48 and in the placebo group of –1.22 to –0.22 [22]. Assuming mean change in T-score in the probiotic group of −0.01 and of −0.72 in the placebo group and a SD of 1.16, a minimum sample size was calculated using Statistica software version 14.0 (Tibco Software Inc., Palo Alto, CA, USA) at 58 patients per group with alpha error 5% and statistical power 90%. After accommodating a data loss of 20%, it was determined that there should be at least 70 women in each study group.

Statistical analyses were performed using Stata version 16.0 (StataCorp LLC., Lakeway, TX, USA). Fisher’s exact test was used for nominal variables (e.g., the occurrence of adverse events and the absence or presence of specific symptoms). For continuous variables, repeated measures ANOVA (RM-ANOVA) or unpaired/paired *t*-tests were used when residuals were normally distributed. For non-normally distributed data, nonparametric two-sample Wilcoxon paired or unpaired signed-rank tests were used. Normal distribution of variables was tested with Shapiro–Wilk test. Data analysis was performed with the use of the per-protocol analysis. A significance threshold of 0.05 was applied for all analyses.

## 3. Results

### 3.1. Patient Characteristics

A total of 172 Caucasian women from Poland were randomized to receive either the probiotic preparation or the placebo. After the 12-month intervention period, two women from the probiotic group and three from the placebo group dropped out due to relocation. Consequently, 167 women (97.1% of the initial cohort) completed the study (Figure 1). Statistical analysis showed no significant differences between the study groups in terms of age, physical development, comorbidities, laboratory test results, BMD and T-scores from DXA scans of the lumbar spine. In both groups, the most common comorbidities were cardiovascular diseases (hypertension and hypercholesterolemia), osteoarthrosis, urinary tract infections, and allergies. The average T-score was 0.19 ± 0.99 in the probiotic group (range: −1.5 to 3.2) and 0.06 ± 1.04 in the placebo group (range: −1.5 to 3.6). The percentage of patients with T-score values above −1.0 SD was similar in both groups, with 83.3% in the probiotic group and 84.3% in the placebo group. The characteristics of the patients are presented in Table 2.

### 3.2. The Effect of Intervention on T-Score of Lumbar Spine

A decrease in T-score of lumbar spine was observed in both groups compared to the baseline values (Table 3). However, in the probiotic group, the reduction did not reach statistical significance, in contrast to the placebo group. The average T-score in the probiotic group decreased from 0.19 ± 0.99 to −0.08 ± 1.05, with no significant trend (*p* = 0.125). In the placebo group, the T-score showed a statistically significant decrease from 0.06 ± 1.04 to −0.28 ± 1.12 (*p* = 0.041). Although there were differences between the probiotic and placebo groups, they did not reach statistical significance after the intervention (*p* = 0.089). The percentage of women with T-scores below −1.5 after 12 months was similar in both groups: 13.1% (*n* = 11) in the probiotic group and 14.5% (*n* = 12) in the placebo group.

### 3.3. The Effect of Intervention on Secondary Outcomes

#### 3.3.1. Serological Markers

**Figure 2:**
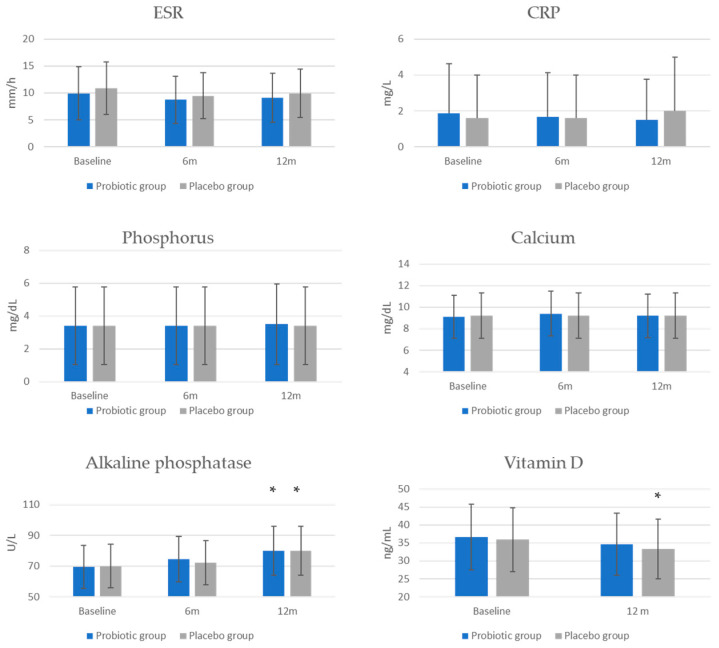
The effect of probiotic intervention on selected serological markers. * *p* < 0.05 in comparison with baseline levels within the group.

Assessment of inflammatory markers (CRP and ESR) as well as calcium and phosphorus concentrations showed no differences after the intervention within or between groups (Figure 2). A statistically significant increase in alkaline phosphatase activity was observed in both groups after 12 months (probiotic group: *p* = 0.0001; placebo group: *p* = 0.018), with no difference between groups. A statistically significant decrease in vitamin D concentration was observed only in the placebo group (*p* = 0.014), with no significant difference between groups. The percentage of women with vitamin D deficiency (<30 ng/mL) was lower in the probiotic group (22.6%, *n* = 19) than in the placebo group (28.9%, *n* = 24), but the difference was not statistically significant.

#### 3.3.2. BMI and Gastrointestinal Symptoms

**Figure 3:**
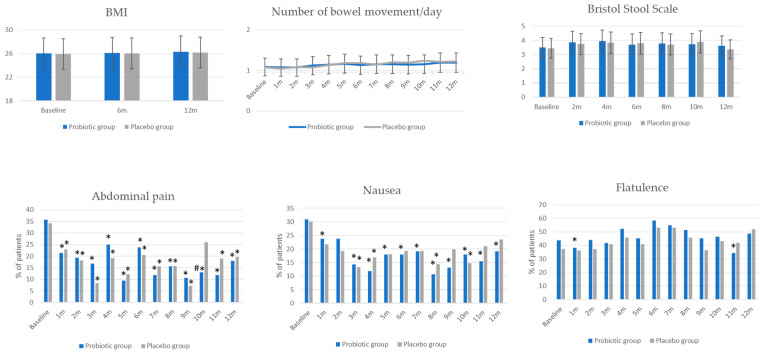
The effect of intervention on BMI and gastrointestinal symptoms. Results for BMI, number of stools per day, and Bristol Stool Scale are presented as mean ± standard deviation. The remaining parameters are presented as a percentage of patients reporting each symptom. * *p* < 0.05 compared to baseline within the group. # *p* < 0.05 between probiotic and placebo groups.

Probiotic supplementation did not affect body weight, and no differences in BMI between groups or within groups during the study period were observed (Figure 3).

Before the intervention, the average number of bowel movements per day was 1.08 ± 0.39 in the probiotic group and 1.07 ± 0.34 in the control group (Figure 3). Stool consistency was normal in 76.2% of individuals in the probiotic group and in 77.1% of individuals in the control group. Abdominal pain was reported by 35.7% of individuals in the probiotic group and in 34.4% in the control group; nausea and flatulence occurred in 30.9% and 30.1% of individuals in the probiotic group, and in 44.0% and 37.3% of individuals in the control group, respectively, with most symptoms being either mild or moderate.

The probiotic intervention had no statistically significant effect on the number of bowel movements or stool consistency (Figure 3). Periodic, statistically significant decreases in intensity of gastrointestinal symptoms were observed in both groups, with no differences between groups, except for a statistically significant increase in the percentage of women without abdominal pain in the 10th month. A statistically significant decrease in nausea and flatulence was more often observed in the probiotic group than in the placebo group compared to baseline (Figure 3).

#### 3.3.3. Satisfaction of the Intervention

**Figure 4:**
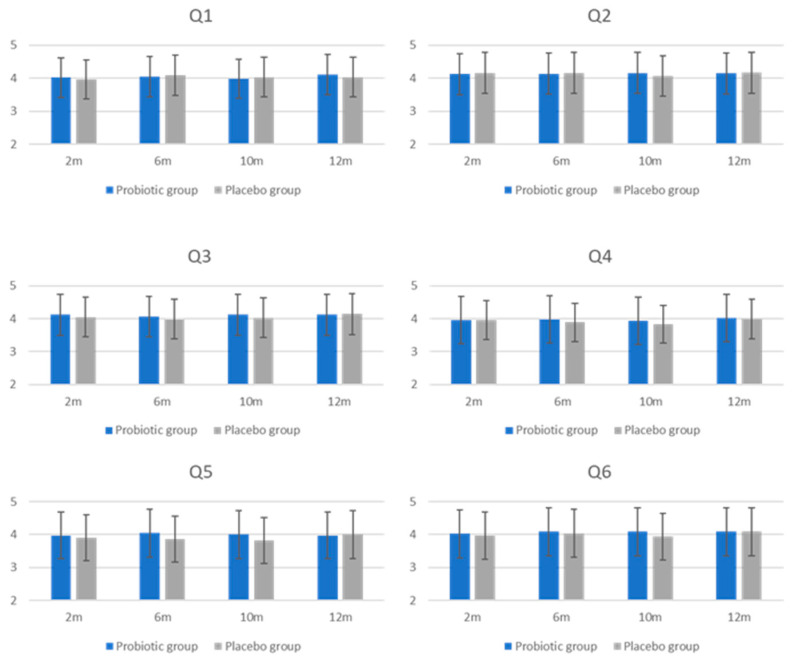
Intervention satisfaction assessment using the Treatment Satisfaction Questionnaire (TSQ). Results are presented as mean ± standard deviation. Satisfaction was assessed after 2 (2 m), 6 (6 m), 10 (10 m), and 12 (12 m) months. Subjects answered 6 questions (Q1–Q6) using a 5-point scale. The *Y*-axis presents scores.

Satisfaction of the intervention, assessed by TSQ after 2, 6, 10, and 12 months, revealed that the majority of respondents in both groups rated their satisfaction as good (4 points) or very good (5 points). For each TQS question, the percentage of women reporting such a score after the intervention was 73.4% and 69.2% for the effectiveness of the preparation (Q1), 77.1% and 80.6% for ease of administration (Q2), 75.8% and 80.6% for recommendations for taking the preparation (Q3), 74.6% and 75.8% for benefits of treatment (Q4), 72.2% and 77.1% for the preponderance of advantages over disadvantages (Q5), and 73.4% and 73.4% for overall satisfaction (Q6) in the probiotic group and in the placebo group, respectively (Figure 4). No statistically significant differences were found between or within groups during the follow-up.

### 3.4. Safety and Tolerance of Probiotic Preparation

**Figure 5:**
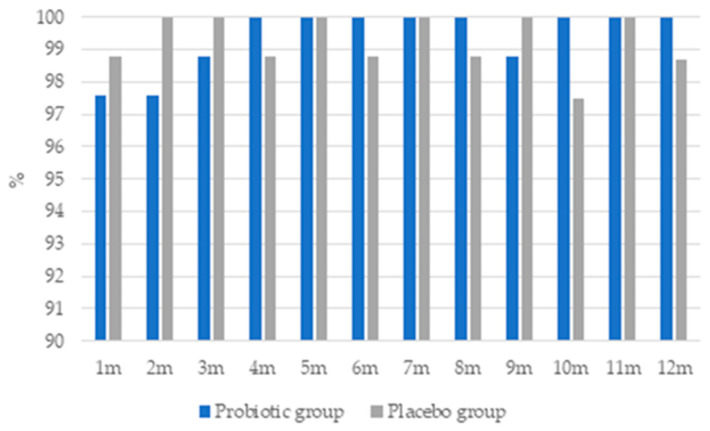
Percentage of patients without adverse events during the 12-month intervention.

Adverse events occurred sporadically in both groups (Figure 5). In the probiotic group, only two (2.4%) women reported adverse events (abdominal pain and headache) after 1 and 2 months of intervention, and only one (1.2%) after 3 and 9 months. In the placebo group, adverse events (abdominal pain, nausea, and headache) were reported by one (1.2%) woman after 1, 4, 6, 8, and 12 months of intervention, and two (2.4%) women after 10 months. No adverse events were reported in the remaining months. There were no statistical differences between groups. In response to Q7 in the TSQ, no woman indicated any adverse events after 2, 6, 10, or 12 months of intervention.

## 4. Discussion

The potential role of gut microbiota in bone health has led to the exploration of probiotics as a strategy to reduce BMD loss during the postmenopausal period [14,15]. While numerous animal model studies have demonstrated the beneficial effects of various probiotic strains on bone mineralization during menopause [17], clinical evidence in postmenopausal women remains less definitive. A 2021 systematic review identified only five randomized, placebo-controlled trials including 497 subjects [23]. The meta-analysis of these trials revealed that probiotics significantly increased BMD in the lumbar spine compared to controls, but showed no significant effects on hip BMD. The authors recommended further studies to confirm these findings. A subsequent systematic review and meta-analysis in 2023, which included six randomized, placebo-controlled trials comprising 632 postmenopausal women, found inconsistent results regarding the impact of probiotics on BMD, with most of them demonstrating no benefit for either the spine or hip bone density [18]. Thus, results of meta-analyses highlight the need for continued investigation into probiotic strains with demonstrable effects on bone health in clinical trials.

In the present randomized, double-blind, placebo-controlled trial including 167 postmenopausal women, we evaluated the effect of *L. paracasei* LPC100 and *L. plantarum* LP140 on lumbar spine mineral density as assessed by DXA-derived T-scores. This study focused on early postmenopausal women (up to 5 years from menopause), a period marked by rapid bone loss. Participants were healthy women with normal serum vitamin D levels (>30 ng/mL) and lumbar spine T-scores above −1.5 SD to explore probiotic potential in preventing bone loss. We present that in the placebo group, a statistically significant reduction in T-score was observed, while in the probiotic group, the decrease did not achieve statistical significance suggesting that probiotic supplementation can reduce the risk of bone loss, although there was no significant differences between study groups (*p* = 0.089). These results align with those of Jansson et al., who reported minimal lumbar spine bone loss in the probiotic group compared to significant loss in the placebo group when supplementing with *L. paracasei* DSM 13434, *L. plantarum* DSM 15312, and *L. plantarum* DSM 15,313 [22]. Other studies have demonstrated that supplementation of 75- to 80-year-old women with low bone density using *Limosilactobacillus* (old name *Lactobacillus*) *reuteri* 6475 for 12 months resulted in significantly reduced bone loss compared to the placebo group [24]. Similarly, beneficial effects on BMD were observed in postmenopausal women supplemented with *Bacillus subtilis* C-3102, which significantly increased total hip BMD compared to the placebo group [25].

Our study is distinct due to its participant selection criteria, being women without osteoporosis and with normal vitamin D levels, who did not require additional vitamin D supplementation. Most prior studies included women with osteopenia or osteoporosis who were often supplemented with vitamin D, calcium, magnesium, or isoflavones [26,27,28], potentially introducing some bias.

By excluding vitamin D supplementation, we could assess the direct effect of pro-biotics on serum vitamin D levels. We found a reduced risk of vitamin D depletion in the blood of women from the probiotic group, while the placebo group exhibited a statistically significant reduction in vitamin D levels. Jones et al. were the first to observe a beneficial effect of probiotic administration on serum levels of fat-soluble vitamins, including vitamin D, in a post hoc analysis of a randomized, controlled trial involving 127 hypercholesterolemic adults [29]. They showed that *Limosilactobacillus reuteri* NCIMB 30,242 administration led to an increase in serum vitamin D after 9 weeks of intake compared to the placebo group. However, the duration of supplementation in their study was relatively short (9 weeks versus 12 months in the current study), and the study groups differed. To our knowledge, the effect of probiotics on serum vitamin D concentration in postmenopausal women has not been described so far.

The role of vitamin D in the maintenance of bone health is well established. Vitamin D deficiency is often associated with bone tissue disorders and is widespread all over the world [30]. Vitamin D influences bone development and calcium homeostasis through its actions in the intestine, kidney, and bone [31]. It regulates calcium absorption in the intestine and kidney by activating transcellular calcium channels that mediate intracellular calcium diffusion. It is suggested that certain probiotics, especially those producing SCFAs, may influence vitamin D metabolism and absorption [11,32]. The strains selected for the current study are potential SCFA producers. The genomes of *L. paracasei* LPC100 and *L. plantarum* LP140 were sequenced revealing that both strains possess complete pathways for the synthesis of acetic acid, with *L. paracasei* LPC100 also having a partial pathway for butyric acid synthesis [33,34]. SCFAs, especially butyric acid, promote intestinal cell growth and increase intestinal absorption areas, enhancing the absorption of vitamin D and other micro- and macro-elements (e.g., calcium and phosphorus) [35]. Butyric acid is also an important factor in osteogenesis induced by parathyroid hormone and plays a major role in the calcium balance in the body [36]. In addition, SCFAs can induce anti-inflammatory responses through the activation of regulatory T lymphocytes [37]. However, similar to other researchers, we did not observe effects of probiotic supplementation on serum levels of inflammation biomarkers (ESR and CRP) or calcium and phosphorus.

As probiotics can affect gut functions, the effect of probiotic supplementation on gastrointestinal symptoms was also assessed in the present study. Despite the lack of differences of gastrointestinal symptoms between the study groups during the intervention, a statistically significant improvement in the severity of some symptoms (abdominal pain and flatulence) was observed more often in the probiotic group compared to the state before the intervention. The positive effects of probiotics on gastrointestinal tract function are well known. Both strains used in the current study, *L. paracasei* LPC100 and *L. plantarum* LP140, had been previously tested in a multi-strain mixture in patients with irritable bowel syndrome (IBS) [38]. The multi-strain probiotic intervention significantly improved IBS symptoms, such as abdominal pain, nausea, and flatulence, and contributed to a significant improvement in quality of life. In the current study, the effect of probiotic intervention between the study groups was not statistically significant, but it should be noted that the study included potentially healthy women, the vast majority of whom did not have gastrointestinal symptoms, and when they did occur, they were mild or moderate.

Recently, Vanitchanont et al. presented that multispecies probiotic administration to postmenopausal women with osteopenia resulted in a significant decrease in serum level of bone resorption marker C-terminal telopeptide of type I collagen after 12 weeks of supplementation compared with baseline (*p*-value = 0.026) [26]. The authors supposed that like experimental animal studies [10,12] probiotics administrated to women were able to induce a slowing down of osteoclast-induced bone resorption. Our clinical study, due to the lack of analyses related to bone turnover markers, does not allow for an explanation of the mechanism of action of administrated probiotics on bone metabolism. We can only speculate that the probiotic strains used in the current study have specific features allowing for an improvement of the intestinal absorption of minerals including calcium, and for modulation of the gut dysbiosis activating pro-inflammatory processes. Probiogenomic analyses of both *L. paracasei* LPC100 [33] and *L. plantarum* LP140 [34] revealed genes encoding SCFAs which can enhance the absorption area of the gut epithelium for compounds necessary for bone mineralization, as well as activate anti-inflammatory cytokines in the gut influencing the gut–bone axis. In addition, the *L. plantarum* LP140 genome [34] revealed genes encoding bacteriocins, which may play a potential role in managing gut dysbiosis in postmenopausal women [39].

### Strengths and Limitations of the Study

The strongest point of the current study is that it is a randomized, double-blind, placebo-controlled clinical trial conducted in three independent centers on a relatively homogeneous group of potentially healthy women in the early postmenopausal period. The study lasted 12 months, and despite the long follow-up period, only 5 of the 172 women did not complete it for reasons unrelated to side effects or lack of satisfaction with the treatment. The scheduled monthly visits and the monitoring of compliance and side effects at each visit are also strengths.

The main limitations of the current study include the lack of assessment of bone turnover biomarkers as well as the composition of the gut microbiome and its metabolome, which would provide a better understanding of the mechanisms related to probiotic supplementation. The limitations of the study also include the relatively short observation period. A 12-month follow-up period is useful, but since bone changes occur slowly, a longer follow-up (e.g., 18 or 24 months) could provide more information on the long-term effects of probiotics on BMD and vitamin D.

## 5. Conclusions

This study suggests that the oral administration of *L. paracasei* LPC100 and *L. plantarum* LP140 from the NORDBIOTIC^TM^ collection may be a viable strategy to prevent bone mineral loss and vitamin D deficiency in postmenopausal women. Twelve-month probiotic supplementation showed potential benefits in maintaining lumbar spine bone demineralization and serum vitamin D levels and in reducing gastrointestinal symptoms. The intervention was safe and well tolerated. The beneficial effects of supplementation may be related to unique features of evaluated strains, including the possibility to produce SCFAs and bacteriocins, but understanding the mechanisms of probiotic action requires further research. Further studies with longer observation periods are also necessary to confirm the beneficial clinical effects of probiotics.

## Figures and Tables

**Table 1:** Specification of NORDBIOTIC™ strains in the evaluated probiotic preparation.

Species	Strain	DSM Number *	CFUs/Capsule
*Lacticaseibacillus paracasei* [*Lactobacillus paracasei*]	LPC100	33793	1.5 × 10^9^
*Lactiplantibacillus plantarum* [*Lactobacillus plantarum*]	LP140	33804	3.5 × 10^9^

* DSM—number given by Leibniz Institute DSMZ—German Collection of Microorganisms and Cell Cultures GmbH; previously used species names are given in square brackets [21]; CFUs = colony-forming units.

**Table 2:** Patient characteristics.

	Probiotic Group (*n* = 84)	Placebo Group (*n* = 83)	*p*-Value between Groups *
Age (years)	56.3 ± 6.8 [56.6; 37.4–69.7]	57.0 ± 7.8 [57.2; 38.2–68.8]	0.896
Height (m)	1.65 ± 0.1 [1.64; 1.49–1.82]	1.64 ± 0.1 [1.64; 1.49–1.95]	0.997
Body weight (kg)	70.54 ± 9.9 [70.5; 48.0–87.0]	70.0 ± 10.0 [69.0; 46.0–88.8]	0.846
BMI	26.0 ± 2.9 [26.4; 18.7–30.0]	25.9 ± 3.3 [26.6; 18.4–30.0]	0.995
Comorbidities	61 (72.6%)	61 (73.5%)	0.899
T-score	0.19 ± 0.99 [0.30; −1.50–3.20]	0.06 ± 1.04 [−0.20; −1.50–3.60]	0.222
Laboratory tests:			
ESR (mm/h)	9.9 ± 7.5 [9.0; 2.0–34.0]	10.9 ± 10.4 [8.0; 2.0–65.0]	0.961
CRP (mg/L)	3.2 ± 4.2 [1.8; 0.1–30.1]	3.0 ± 4.8 [1.6; 0–38.7]	0.198
Ca (mg/dL)	9.1 ± 2.4 [10.1; 7.7–10.3]	9.4 ± 2.2 [10.3; 8.1–10.4]	0.914
P (mg/dL)	3.9 ± 0.7 [3.4; 1.0–5.2]	3.3 ± 0.6 [3.4; 1.1–4.6]	0.662
Vitamin D (ng/mL)	41.1 ± 12.3 [36.6; 31.0–93.0]	40.2 ± 12.2 [35.9; 31.0–83.4]	0.471
Alkaline phosphatase (U/L)	71.5 ± 19.8 [69.5; 34–124]	74.6 ± 26.0 [70.0; 23.0–161]	0.680

The results are presented as mean ± standard deviation; the median and the lowest and highest values are shown in square brackets. * Due to the non-normal distribution, statistical analysis of differences in continuous variables between groups was performed using the Wilcoxon signed-rank test. Normal distribution was assessed with the Shapiro–Wilk test. Comorbidities are shown as number of women (%). ESR = erythrocyte sedimentation rate; BMI = body mass index; SD = standard deviation; DXA = dual-energy X-ray absorptiometry; CRP = C-reactive protein; Ca = calcium, P = phosphorus. Normal values: ESR < 30 mm/h; CRP < 5 mg/L; Ca = 9.0–11.0 mg/dL; P = 2.8–5.0 mg/dL; vitamin D > 30 ng/mL; alkaline phosphatase 30–120 U/L.

**Table 3:** The effect of probiotic intervention on T-score from lumbar spine DXA scan.

Groups	T-Score at Baseline		T-Score after 12-Month Intervention		*p*-Value	
	Mean ± SD	Median [Range]	Mean ± SD	Median [Range]	Within Groups	Between Groups
Probiotic (*n* = 84)	0.19 ± 0.99	0.3 [−1.5–3.2]	−0.08 ± 1.05	0 [−2.9–1.9]	0.125	0.089
Placebo (*n* = 83)	0.06 ± 1.04	−0.2 [−1.5–3.6]	−0.28 ± 1.12	−0.4 [−2.4–3.9]	0.041	

SD = standard deviation.

## Data Availability

The description of the protocol study is available at “https://clinicaltrials.gov/study/NCT06375668” (accessed on 1 October 2024). The data presented in this study are available on request from the corresponding author. The data are not publicly available due to privacy protection.
